# A functional variant rs12904 in the miR-200c binding site was associated with a decreased risk of ischemic stroke

**DOI:** 10.1186/s12944-019-1060-1

**Published:** 2019-05-10

**Authors:** Zhi-Neng Zeng, Ling-Ling Liu, Yong-Ling He, Xiang Shi, Ye-Sheng Wei

**Affiliations:** 1 Department of Clinical Laboratory, Affiliated Hospital of Guilin Medical University, Guilin, Guangxi China; 2 Department of Neonatology, Affiliated Hospital of Guilin Medical University, Guilin, Guangxi China

**Keywords:** miR-200c, Genome-wide association study, Polymorphism, Ischemic stroke

## Abstract

Genome-wide association study (GWAS) identified chromosome 12p13 rs12425791 and rs11833579 as susceptibility loci of ischemic stroke (IS) in a European population. However, conflicting results were obtained in subsequent replication analysis. miR-200c, located on chromosome 12p13, was found to have a neuroprotective effect on ischemia. Our aim of this study was to investigate the association of the rs12425791, rs11833579 and rs12904 in the binding site of miR-200c with the risk of IS. The rs12425791, rs11833579, and rs12904 were genotyped using a TaqMan allelic discrimination assay. The results were verified by Sanger sequencing. We found that the rs12904 AG/GG genotypes and G allele were associated with a decreased risk of IS (AG/GG vs. AA: adjusted OR = 0.64; 95% CI, 0.44–0.95; G vs. A: adjusted OR = 0.65; 95% CI, 0.46–0.93). The combined genotypes of the rs11833579AG/AA and rs12904AG/GG were also associated with a reduced risk of IS (OR = 0.65; 95% CI, 0.46–0.93). These findings suggest that the rs12904 may have a jointly protective effect against the risk of IS.

## Introduction

Stroke is a major cause of death and disability worldwide, and about 73–87% of strokes are ischemic [1–3]. It is evident that ischemic stroke (IS) has a substantial genetic component, especially in patients less than 70 years of age [4–7]. For example, family history is a risk factor for stroke, and monozygotic twins are more likely to be concordant than dizygotic twins [8]. Our previous work showed that *S100B* rs9722, growth differentiation factor-15 (*GDF-15*) rs1804826, and miR-143/145 rs4705342 were genetic risk factors for the occurrence of IS, probably by affecting the expression levels of serum S100B, soluble GDF-15, and miR-145 [9–11].

Over the past years, microRNAs (miRNAs), have been identified as important gene regulators in the development of human diseases including IS by binding to the 3′-untranslated region (3’UTR) of target mRNAs [12–14]. Among them, miR-200c was differentially expressed and had a neuroprotective effect on ischemia, indicating that miR-200c may be used as a potential target for therapeutic intervention [13–15].

In 2009, genome-wide association study (GWAS) identified that single nucleotide polymorphisms (SNPs) on chromosome 12p13 (i.e., rs12425791 and rs11833579) were associated with the risk of stroke in a Dutch population [16]. A replication study performed in a Swedish population, however, did not confirm the finding of the rs12425791 conferring the substantial risk for IS [17]. In a Chinese Han population, the results were conflicting. Tong et al. reported that the rs11833579A allele may play a role in mediating susceptibility and occurrence to IS [18, 19], while Ding et al. reported no evidence for the association of 12p13 SNPs rs11833579 and rs12425791 with IS risk [20]. Additionally, an rs12904 A allele in the 3’UTR of *EFNA1* disrupted the binding site of miR-200c that located on chromosome 12p13, resulting in translational repression and elevated levels of *EFNA1* [21]*.* Based on this background, we hypothesized that the 3 SNPs on chromosome 12p13 were related to the risk of IS. In the current study, we performed a case-control study to evaluate whether the 3 SNPs were risk factors for the etiology of IS.

## Materials and methods

### Study population

**Fig. 1:**
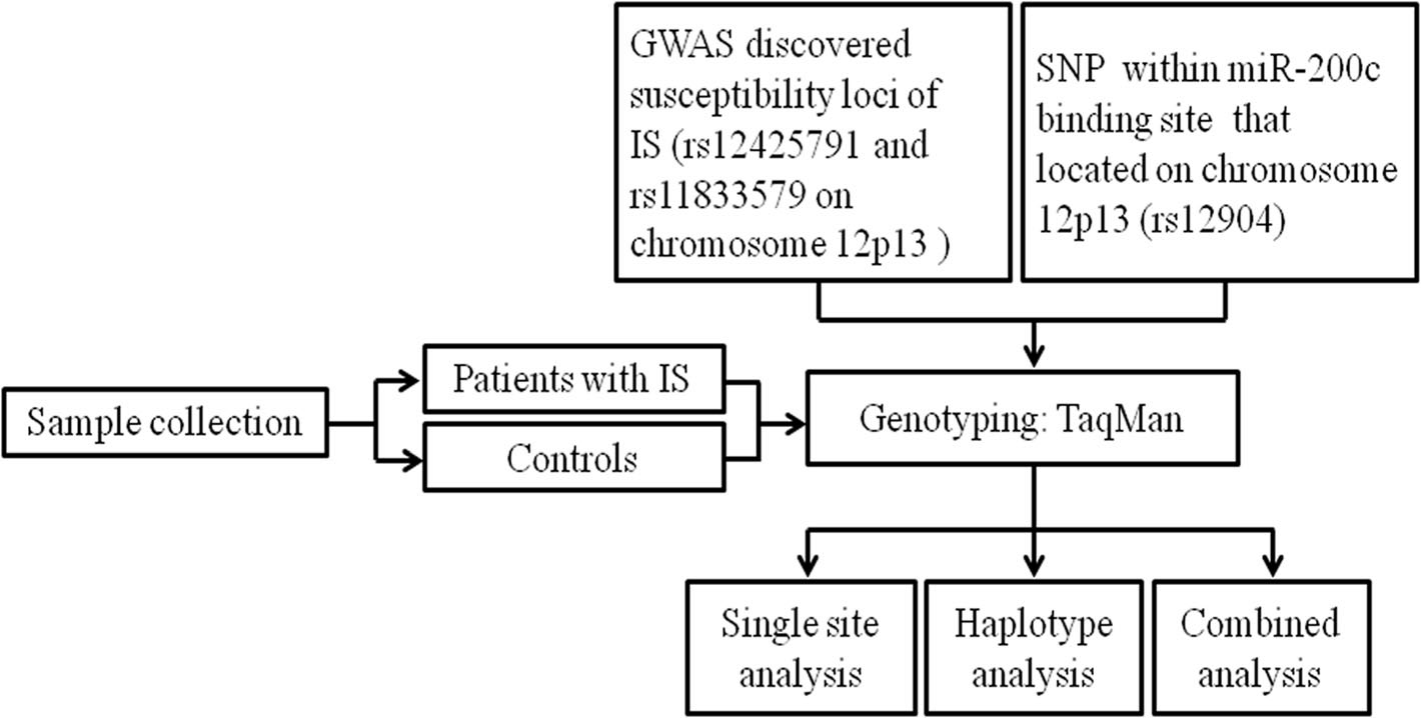
Flow chart of the study. GWAS, genome-wide association study; IS, ischemic stroke; SNP, single nucleotide polymorphisms

The study protocol was approved by the Review Board of the Affiliated Hospital of Youjiang Medical University for Nationalities. All subjects signed informed consent to participate in the study. The flow chart of the study is shown in Fig. 1. The study subjects included 328 patients with IS and 331 controls who were collected from the Affiliated Hospital of Youjiang Medical University for Nationalities, Guangxi, China between January 2013 and September 2016. Detailed information of the study population was described in our previous work [9]. Briefly, IS was defined as an acute focal or global neurologic deficit that persisted for more than 24 h. IS diagnosis was confirmed by clinical symptoms, physical examinations and cranial computed tomography or magnetic resonance imaging. Patients with hemorrhagic stroke, traumatic brain injuries, cardiogenic thrombosis, brain tumors, and family history of stroke were excluded. Controls were enrolled from the Health Medical Center of the hospital during the same period. Those who had brain tumors, autoimmune diseases, haematological disorder, and family history of stroke were excluded. All the cases and controls were unrelated Han Chinese who resided in Guangxi province. Clinical data, such as age, gender, total cholesterol (TC), triglyceride (TG), high-density lipoprotein cholesterol (HDL-C), low-density lipoprotein cholesterol (LDL-C), very low-density lipoprotein cholesterol (VLDL-C), apolipoprotein A1 (Apo-A1), and apolipoprotein B (Apo-B) were obtained from medical record of the hospital.

### Genotyping

Genomic DNA was extracted from peripheral blood samples using the DNA extraction kit (Qiagen, Valencia, CA, USA). The chromosome 12p13 SNPs were genotyped using a TaqMan allelic discrimination assay on an ABI 7900HT analyzer (Applied Biosystems, CA, USA). The SNP assay ID of rs12425791, rs11833579, and rs12904 was C__12094896_10, C__1665834_10, and C__191594_10, respectively. Approximately, 5% of all samples were randomly selected to be verified by Sanger sequencing, and the results were 100% consistent.

### Statistical analysis

The chromosome 12p13 SNPs were tested for Hardy–Weinberg equilibrium (HWE) among cases and controls using the chi-squared test. Continuous data were presented as mean ± standard deviation (SD) and compared using the Student’s *t*-test, while discrete data were presented as frequencies (percentages) and compared using the χ^2^ test. Odds ratio (OR) with 95% confidence interval (CI) were used to assess the association between chromosome 12p13 SNPs and IS risk after adjustments for age, gender, hypertension, type 2 diabetes, and smoking using multivariate logistic regression. Linkage disequilibrium (LD) and haplotype analysis were performed using an online software SHEsis (http://analysis.bio-x.cn/myAnalysis.php) [22]. Statistical analysis was performed using SPSS version 17.0 software (SPSS, Chicago, IL, USA). A *P* value < 0.05 was considered as statistically significant.

## Results

### Characteristics of the study population

The distributions of the demographic and clinical characteristics of the cases and controls are presented in Table 1. No significant difference was observed in age, gender, cigarette smoking, and TC levels between cases and controls. When compared to controls, IS patients had significantly higher levels of TG, LDL-C, VLDL-C, and Apo-B and lower levels of HDL-C and Apo-A1 (*P* <  0.001).

**Table 1:** Baseline characteristics of the study population

Variables	Controls, *n* = 331	Patients with IS, *n* = 328	*P* value
Age, years (± SD)	60.8 (± 11.2)	62.0 (± 11.4)	0.18
Male/ Female (%)	217 (65.6)/ 114 (34.4)	232 (70.7)/ 96 (29.3)	0.15
Hypertension, yes/no (%)	62 (18.7)/269 (81.3)	172 (52.4)/156 (47.6)	< 0.001
Type 2 diabetes, yes/no (%)	26 (7.9)/305 (92.1)	47 (14.3)/281 (85.7)	0.008
Smoking, yes/no (%)	86 (26.0)/245 (74.0)	101 (30.8)/227 (69.2)	0.17
TC, mmol/L	4.77 ± 0.78	4.67 ± 1.13	0.19
TG, mmol/L	1.33 ± 0.80	1.82 ± 1.57	< 0.001
HDL-C, mmol/L	1.52 ± 0.39	1.14 ± 0.33	< 0.001
LDL-C, mmol/L	2.34 ± 0.99	2.94 ± 0.95	< 0.001
VLDL-C, mmol/L	0.68 ± 0.50	0.85 ± 0.69	< 0.001
Apo-A1, g/L	1.77 ± 1.09	1.24 ± 0.26	< 0.001
Apo-B, g/L	0.76 ± 0.30	1.00 ± 0.30	< 0.001

*IS* ischemic stroke, *SD* standard deviation, *TC* total cholesterol, *TG* triglyceride, *HDL-C* high-density lipoprotein cholesterol, *LDL-C* low-density lipoprotein cholesterol, *VLDL-C* very low-density lipoprotein cholesterol, *Apo-A1* apolipoprotein A1; *Apo-B* apolipoprotein B

### Main effect of chromosome 12p13 SNPs on IS risk

Genotype distributions of the chromosome 12p13 SNPs in cases and controls did not deviate from HWE (*P* > 0.05 for all loci). Table 2 displays the genotype and allelic frequencies of the three SNPs between cases and controls. The AG/GG genotype frequency of the rs12904 was 20.1% in cases and 28.4% in controls, and the *P* value of 0.03 after adjusting for age, gender, hypertension, type 2 diabetes, and smoking (OR = 0.64; 95% CI, 0.44–0.95). The frequency of the rs12904 G allele was 11.0% in cases and 16.0% in controls, and the *P* value of 0.02 after adjusting for age, gender, hypertension, type 2 diabetes, and smoking (OR = 0.65; 95% CI, 0.46–0.93). The other two loci (rs12425791 and rs11833579) showed no significant differences between IS cases and controls in either genotype or allelic analysis.

**Table 2:** Associaiton between chromosome 12p13 SNPs and risk of IS

Genotypes	Controls, *n* = 331 (%)	IS, *n* = 328 (%)	Adjusted OR (95% CI) ^a^	Adjusted *P* value ^a^
rs12425791				
GG	184 (55.6)	172 (52.4)	1.00	
AG/AA	147 (44.4)	156 (47.6)	1.02 (0.73–1.42)	0.90
G	486 (73.4)	476 (72.6)	1.00	
A	176 (26.6)	180 (27.4)	1.00 (0.76–1.29)	0.97
rs11833579				
GG	161 (48.6)	171 (52.1)	1.00	
AG/AA	170 (51.4)	157 (47.9)	0.89 (0.64–1.24)	0.49
G	452 (68.3)	467 (71.2)	1.00	
A	210 (31.7)	189 (28.8)	0.89 (0.69–1.14)	0.35
rs12904				
AA	237 (71.6)	262 (79.9)	1.00	
AG/GG	94 (28.4)	66 (20.1)	0.64 (0.44–0.95)	0.03
A	556 (84.0)	584 (89.0)	1.00	
G	106 (16.0)	72 (11.0)	0.65 (0.46–0.93)	0.02

*SNP* single nucletide polymorphism, *IS* ischemic stroke, *OR* odds ratio, *CI* confidence interval. ^a^Adjusted by age, gender, hypertension, type 2 diabetes, and smoking

### Haplotype analysis

The LD measurement and haplotype construction were conducted in the current study. As shown in Table 3, the G-A-G haplotype had a trend to decrease the susceptibility of IS compared to the G-G-A haplotype. The difference, however, did not reach the significance, with the *P* value of 0.06 (OR = 0.53; 95% CI, 0.28–1.02).

**Table 3:** Haplotype analysis of chromosome 12p13 SNPs with IS risk

rs12425791	rs11833579	rs12904	Controls (%)	IS (%)	OR (95% CI)	*P* value
G	G	A	286 (43.2)	311 (47.4)	1.00	
G	A	A	132 (19.9)	120 (18.3)	0.84 (0.62–1.12)	0.23
A	G	A	97 (14.7)	106 (16.2)	1.01 (0.73–1.38)	0.98
A	A	A	41 (6.2)	47 (7.2)	1.05 (0.67–1.65)	0.82
G	G	G	42 (6.3)	30 (4.6)	0.66 (0.40–1.08)	0.10
A	G	G	27 (4.1)	20 (3.0)	0.68 (0.37–1.24)	0.21
G	A	G	26 (3.9)	15 (2.3)	0.53 (0.28–1.02)	0.06
A	A	G	11 (1.7)	7 (1.1)	0.59 (0.22–1.53)	0.27

*SNP* single nucletide polymorphism, *IS* ischemic stroke, *OR* odds ratio, *CI* confidence interval

### Combined analysis

Since the rs12904 AG/GG genotypes had a protective role against the risk of IS in single SNP association analysis, we evaluated whether rs12425791- rs12904 and rs11833579- rs12904 had combined effects on the risk of IS. As shown in Table 4, the frequencies of the combined genotypes of rs11833579AG/AA and rs12904AG/GG were 10.1% in cases and 7.6% in controls, with the *P* value of 0.02 (OR = 0.56; 95% CI, 0.34–0.93).

**Table 4:** Combined effects of chromosome 12p13 SNPs on the risk of IS

Combined genotypes	Controls (%)	IS (%)	OR (95% CI)	*P* value
rs12425791- rs12904				
rs12425791GG + rs12904AA	136 (41.1)	141 (43.0)	1.00	
rs12425791GG + rs12904AG/GG	48 (14.5)	31 (9.5)	0.62 (0.37–1.04)	0.07
rs12425791AG/AA + rs12904AA	101 (30.5)	121 (36.9)	1.16 (0.81–1.65)	0.42
rs12425791AG/AA + rs12904AG/GG	46 (13.9)	35 (10.7)	0.73 (0.45–1.21)	0.22
rs11833579- rs12904				
rs11833579GG + rs12904AA	117 (35.3)	138 (42.1)	1.00	
rs11833579GG + rs12904AG/GG	44 (6.7)	33 (10.1)	0.64 (0.38–1.06)	0.08
rs11833579AG/AA + rs12904AA	120 (36.3)	124 (37.8)	0.88 (0.62–1.25)	0.46
rs11833579AG/AA + rs12904AG/GG	50 (7.6)	33 (10.1)	0.56 (0.34–0.93)	0.02

*SNP* single nucletide polymorphism, *IS* ischemic stroke, *OR* odds ratio, *CI* confidence interval

## Discussion

In the current study, we evaluated the association between the rs12904 in the miR-200c binding site and IS risk. As miR-200c located on chromosome 12p13 that is a susceptibility loci of IS, we also performed a replication analysis of the 12p13 SNPs (i.e., rs11833579 and rs12425791) with the risk of IS. We found a significant difference in the distributions of the rs12904 AG/GG genotypes and G allele between cases and controls. Results from combined analysis showed that the combined genotypes of the rs11833579AG/AA and rs12904AG/GG decreased the risk of IS. These findings implicate that the rs12904 may be used as a biomarker for the etiology of IS.

Previously, GWAS identified 2 intergenic SNPs (i.e., rs12425791 and rs11833579) on chromosome 12p13, which contributed to the risk of IS in a Dutch population [16]. Subsequent studies, however, obtained conflicting results. Matsushita et al. reported that the rs12425791 was significantly associated with atherothrombotic stroke in a Japanese population [23], whereas Olsson et al. reported that the rs12425791 did not confer a substantial risk for IS in a Swedish population [17]. The conflicting results may not be explained by different ethnicities because inclusive results were also observed even in the same Chinese Han population. Wang et al. reported that the rs12425791 A was a risk allele for IS [24], while Tong et al. reported that the rs12425791 was not a risk factor for IS [18, 25]. Due to the limited samples of 182 cases and 66 controls, the results reported by Wang and colleagues may occur by chance. Meta-analysis was then performed to provide more precise data. Nevertheless, conflicting results were also found. In 2012, evidence from meta-analysis revealed that the rs12425791 was significantly associated with the risk of IS under a dominant genetic model [26, 27]. In contrast, an updated meta-analysis carried out in 2013 showed that no significant association between the rs12425791 and IS risk [28]. Similar to the negative data, we found in this study that the rs12425791 did not confer a substantial risk for IS in the Chinese Han population.

Regarding the rs11833579, some authors reported that the AA genotype increased the risk of IS [18, 29], while some authors reported an absence of association with IS [20, 23–25, 30]. Consistent with the null results, in this study, we failed to find any association of the rs11833579 with IS risk. Some possibilities may be used for explaining the inconclusive results. All the study design was hospital-based, and the selection bias of controls cannot be ruled out. Moreover, gene-environment interaction may be a key event in the development of IS. Further population-based studies are required to confirm the results.

Since GWAS-discovered IS risk loci (rs12425791 and rs11833579) were not verified by our results, we speculated that potentially functional SNPs within chromosome 12p13 may contribute to the risk of IS. miR-200c, located on chromosome 12p13, was found to be upregulated after ischemia in animal model [13–15]. Reduction of miR-200c can protect the brain from transient focal cerebral ischemia by targeting reelin or prolyl hydroxylase 2 [13, 14]. Previously, an SNP rs12904 was found to be functional, with the G > A change leading to altered regulation of luciferase expression and *EFNA1* mRNA levels [21]. We therefore hypothesized that the rs12904 may be a risk factor for the pathogenesis of IS. Our findings confirmed this hypothesis. We found that the rs12904AG/GG genotypes had a 0.63-fold decreased risk of IS. Notably, we found that carriers with the combined genotypes of rs11833579AG/AA and rs12904AG/GG had a 0.56-fold decreased risk of IS. The more effective effect of the combined genotypes confirmed the idea that IS cannot be attributed to a single gene.

We have to admit some limitations in this study. The study design was hospital-based and there are possibilities of selection bias of study population. Most patients received lipids lowering treatments, which may influence the results in this study. It is demonstrated that nutraceuticals and functional food ingredients may reduce the incidence of stroke [31–33]. In this study, however, these environmental factors were not available, and thus gene-environment interaction cannot be performed. Further studies solving these limitations are needed.

## Conclusion

In conclusion, this is the first study reporting that the rs12904 AG/GG genotypes were associated with a reduced risk of IS. These findings suggest that the rs12904 in the miR-200c binding site may act as a biomarker for the development of IS in the Chinese population. Further studies are of great importance to understand the biologic function of the rs12904 in the progression of IS.

## Abbreviations

3’UTR: 3′-untranslated region
Apo-A1: Apolipoprotein A1
Apo-B: Apolipoprotein B
CIs: Confidence intervals
GDF-15: growth differentiation factor-15
GWAS: Genome-wide association study
HDL-C: High-density lipoprotein cholesterol
HWE: Hardy Weinberg equilibrium
IS: Ischemic stroke
LD: Linkage disequilibrium
LDL-C: Low-density lipoprotein cholesterol
miRNAs: microRNAs
OR: Odds ratio
SD: Standard deviation
SNPs: Single nucleotide polymorphisms
TC: Total cholesterol
TG: Triglyceride
